# Comparative efficacy of three Bayesian variable selection methods in the context of weight loss in obese women

**DOI:** 10.3389/fnut.2023.1203925

**Published:** 2023-07-18

**Authors:** Nicola Pesenti, Piero Quatto, Elena Colicino, Raffaella Cancello, Massimo Scacchi, Antonella Zambon

**Affiliations:** ^1^Department of Statistics and Quantitative Methods, Division of Biostatistics, Epidemiology and Public Health, University of Milano-Bicocca, Milan, Italy; ^2^Department of Economics, Management and Statistics, University of Milano-Bicocca, Milan, Italy; ^3^Department of Environmental Medicine and Public Health, Icahn School of Medicine at Mount Sinai, New York, NY, United States; ^4^Obesity Unit and Laboratory of Nutrition and Obesity Research, Department of Endocrine and Metabolic Diseases, IRCCS Istituto Auxologico Italiano, Milan, Italy; ^5^Division of Endocrinology and Metabolic Diseases, IRCCS Istituto Auxologico Italiano, Milan, Italy; ^6^Department of Clinical Science and Community Health, University of Milan, Milan, Italy; ^7^Istituto Auxologico Italiano, IRCCS, Biostatistic Unit, Milan, Italy

**Keywords:** variable selection, correlated exposures, Bayesian kernel machine regression, Bayesian semiparametric regression, Bayesian least absolute shrinkage and selection operator, obesity

## Abstract

The use of high-dimensional data has expanded in many fields, including in clinical research, thus making variable selection methods increasingly important compared to traditional statistical approaches. The work aims to compare the performance of three supervised Bayesian variable selection methods to detect the most important predictors among a high-dimensional set of variables and to provide useful and practical guidelines of their use. We assessed the variable selection ability of: (1) Bayesian Kernel Machine Regression (BKMR), (2) Bayesian Semiparametric Regression (BSR), and (3) Bayesian Least Absolute Shrinkage and Selection Operator (BLASSO) regression on simulated data of different dimensions and under three scenarios with disparate predictor-response relationships and correlations among predictors. This is the first study describing when one model should be preferred over the others and when methods achieve comparable results. BKMR outperformed all other models with small synthetic datasets. BSR was strongly dependent on the choice of its own intrinsic parameter, but its performance was comparable to BKMR with large datasets. BLASSO should be preferred only when it is reasonable to hypothesise the absence of synergies between predictors and the presence of monotonous predictor-outcome relationships. Finally, we applied the models to a real case study and assessed the relationships among anthropometric, biochemical, metabolic, cardiovascular, and inflammatory variables with weight loss in 755 hospitalised obese women from the Follow Up OBese patients at AUXOlogico institute (FUOBAUXO) cohort.

## Highlights

- We compared the variable selection ability of three Bayesian variable selection models: Bayesian Kernel Machine Regression (BKMR), Bayesian Semiparametric Regression (BSR) and Bayesian Least Absolute Shrinkage and Selection Operator (BLASSO) using both simulated and real data.
- For large sample size data, BKMR and BSR may be employed indiscriminately, as they were able to capture complex relationships between predictors and the outcome even in presence of highly correlated variables.
- For small sample size data: BKMR correctly selected the important variables, while BSR was strongly dependent on the choice of its tuning parameter and additional considerations should be taken into account to ensure an accurate variable selection.
- BLASSO should be preferred only when it is reasonable to hypothesise the absence of interactions between predictors and in the presence of monotonous relationships.
- In general, the performance of these models strongly depends on the sample size of the dataset, the correlation structure, and the predictor-outcome relationships.

## Introduction

The advent of exposomics and the abundance of novel toxicological information have made the identification of the associations between both exposures and their molecular responses and a health outcome more challenging. Therefore, many variable selection approaches have increased in importance and popularity (1). When multiple exposures and their molecular responses co-occur and have a strong complex correlation structure, traditional statistical models are limited in accounting for multicollinearity or standard error inflation (2). To reduce this problem, dimensionality reduction methods—such as principal component and factor analyses—become very valuable. However, those approaches focus on the transformation of the original variables thus leading to interpretability issues. In addition, multiple co-occurring predictors can have nonlinear and nonadditive relationships with the health outcome, and most statistical methods fail to properly model those relationships. Penalised regression methods are used in this context, such as the least Absolute Shrinkage and Selection Operator (LASSO) (3) and its numerous variants (4–7), along with Bayesian variable selection methods, which have recently been developed to handle jointly multiple correlated predictors and both nonlinear and nonadditive relationships, allowing for the inclusion of prior information (8, 9).

Among these Bayesian methods, those employing spike-and-slab priors (10) or shrinkage priors (11) stand out for features selection. These methods are now widely studied and employed within the environmental epidemiological literature (9, 12), but only a few studies have evaluated the differences and similarities of those approaches in a more general setting.

In this work, we compared three supervised Bayesian models: the Bayesian Kernel Machine Regression (BKMR) (13), the Bayesian Semiparametric Regression (BSR) (14), and the Bayesian LASSO (BLASSO) (15) and we provided useful and practical guidelines of their use. BKMR models the outcome-predictors associations through the use of a kernel function of predictors, while BSR flexibly models the relationships between the predictors and the outcome by employing natural splines. We evaluated the models’ goodness of fit and selection results, simulating several predictors with a complex correlation structure and with disparate relationships with a continuous outcome and considering data with different sample sizes. We finally assessed the models’ performance on a real case study. We leveraged data on weight loss in hospitalised obese women from the Follow Up OBese patients at AUXOlogico institute (FUOBAUXO) cohort (16) and determined the association between biochemical, anthropometric, and clinical variables on weight loss percentage in these patients over a period of 40 days. The paper is organised as follows. In Section 2, we provide a brief overview of the models; in Section 3, we describe the simulation studies for evaluating the models’ selection and performance based on three scenarios; in Section 4, we illustrate the methods’ performance on data from the FUOBAUXO study; and in the last section, we offer some concluding remarks and suggestions for further research.

## Methods

In this section we summarise the methodologies applied throughout the study. We assume a continuous outcome variable Y and several main predictors Z and confounders/covariates X for all models. The BKMR model has the following form:

(1) $$Y_{i}=h\left(z_{i1},\ldots ,z_{iM}\right)+x_{i}^{\prime }\beta +\varepsilon_{i}$$

where Y_i_ is the outcome for individual i(i=1,…,n) ; z_im_ is the m^th^ predictor, also called the exposure variable; x_i_ is a vector of potential confounders; h denotes the unknown exposure-response function to be estimated; β represents the effect of the covariates; and ε_i_ represents residuals iid N(0, σ^2^). To model the h function, BKMR uses a Gaussian kernel machine representation of the form:

(2) $$h\left(Z_{i}\right)=\alpha_{0}+\overset{n}{\underset{j=1}{\sum }}\alpha_{j}K\left(Z_{i},Z_{j}\right)$$

where $Z_{i}={\left(z_{i1},\ldots {\mathrm{,z}}_{\mathrm{iM}}\right)}^{T}$ is the vector of predictors’ values for the individual i, K(.) is the Gaussian kernel, and $\alpha_{i}$ represents unknown parameters (13). Such representation can include a large number of variables of interest and allows for nonlinear and nonadditive relationships between these variables and the health outcome. In linear models, including linear or spline terms of all main predictors and their interactions with the outcome can lead to over-fitting issues due to the high number of parameters to estimate. The use of the kernel machine representation for h addresses this concern by regularising the high-dimensional response function, with the result that subjects with similar exposures will have similar health risks (13). Prior distributions on all the unknown parameters are placed as described in the supplemental material of (13). Adding an auxiliary parameter to the kernel function, which assumes a value of zero when the predictor is no longer included in the model, and a value of one otherwise, BKMR allows for variable selection. The posterior inclusion probabilities (PIPs), which provide measures of variable importance, are estimated for each variable included in the model. Finally, the interactions can be studied using a graphic tool, investigating the predictor-response function of a single predictor in Z for another predictor in Z fixed at various quantiles (and for the remaining predictors set to a particular value). The BSR model is similar to the form of BKMR (1), but the predictor-response function h is modelled through the use of suitable spline functions:

(3) $$h\left(z_{i1},\ldots ,z_{iM}\right)=\overset{M}{\underset{j=1}{\sum }}f_{j}\left(z_{ij}\right)+\underset{j<K}{\sum }f_{jk}\left(z_{ij},z_{ik}\right)+\ldots ,$$

where each function *f*(.) is a natural cubic spline of a required dimension d, and dots indicate that the summation extends all the way through M-way interactions (14). The d parameter corresponds to the number of degrees of freedom used to model the effects of the predictors. The authors suggest building the model for different d values and using the Watanabe–Akaike information criterion (WAIC) to evaluate which model should be utilised going forward.

As a result of variable selection, the BSR provides the PIPs for the main effects and the interactions. Lastly, the LASSO is a technique for improving the ordinary least squares estimates for a linear exposure-response function h by imposing the L1 penalty on the regression coefficients, shrinking or setting them to zero:

(4) $${\hat{\alpha }}_{lasso}=\arg\underset{\alpha }{\min}\left[\overset{n}{\underset{i=1}{\sum }}{\left(y_{i}-\alpha_{0}-\overset{M}{\underset{j=1}{\sum }}\alpha_{j}z_{ij}\right)}^{2}+\lambda \overset{M}{\underset{j=1}{\sum }}\left|\alpha_{j}\right|\right]$$

where $\alpha =\left(\alpha_{0},\alpha_{1},\ldots ,\alpha_{M}\right).$ The λ parameter controls the amount of shrinkage that is applied to the estimates. Within a Bayesian context, BLASSO is obtained by placing a conditional Laplace prior on the α parameter (15), considering that LASSO estimates can be interpreted as posterior mode estimates when the regression parameters have independent and identical Laplace priors (3).

### Software

The R programming language was used for all analyses, along with open-source software packages for BKMR (*bkmr*), BSR (*NLinteraction*), and BLASSO (*monomvn*).

### Simulation

We compared the BKMR, BSR, and BLASSO models in terms of their ability with feature selection and performance prediction in three simulated scenarios with: (1) linear, (2) quadratic, and (3) logistic predictor-response relationships. We considered each scenario under two circumstances: with and without interactions among predictors. Changes in correlations structures and sample size (small, *n* = 100; large, *n* = 1,000) were also evaluated, and two prior settings were used in each model.

**Figure 1:**
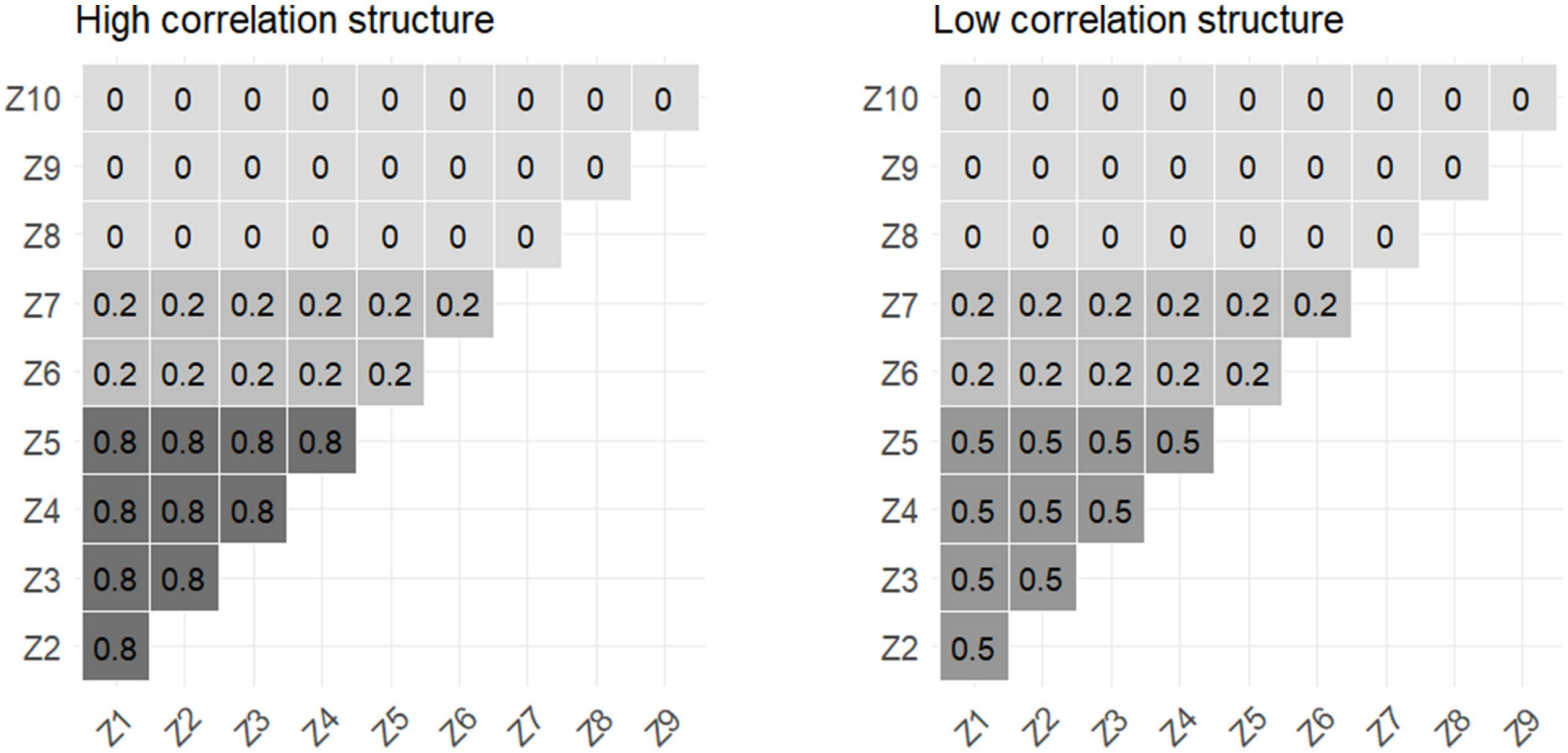
High- and low-correlation structure for the simulated datasets.

The correlation structures were set as moderate (*r* = 0.5) or high (*r* = 0.8) between the first five predictors and low (*r* = 0.2) or absent between all the others (Figure 1).

We assessed the robustness of each model by changing the underlying priors in two situations: for BKMR and BSR, we assumed that a mixture component z_m_ was included in the model with a prior probability π following a beta(a_π_ = 2, b_π_ = 2) or beta(a_π_ = 2, b_π_ = 6) distribution such that, *a priori*, we would expect, respectively, 50 and 25% of the components to be included (6, 7). For *λ^2^* (square of the lasso penalty parameter) of BLASSO, we used gamma distribution priors with α (shape) and β (rate) parameters (1, 0.5) and (1, 2), corresponding to exponential distributions (10). A total of 48 configurations (*x*3 scenarios, *x2* interaction situations, *x2* correlation structures, *x2* sample sizes, *x2* prior settings) were studied for all the models.

**Figure 2:**
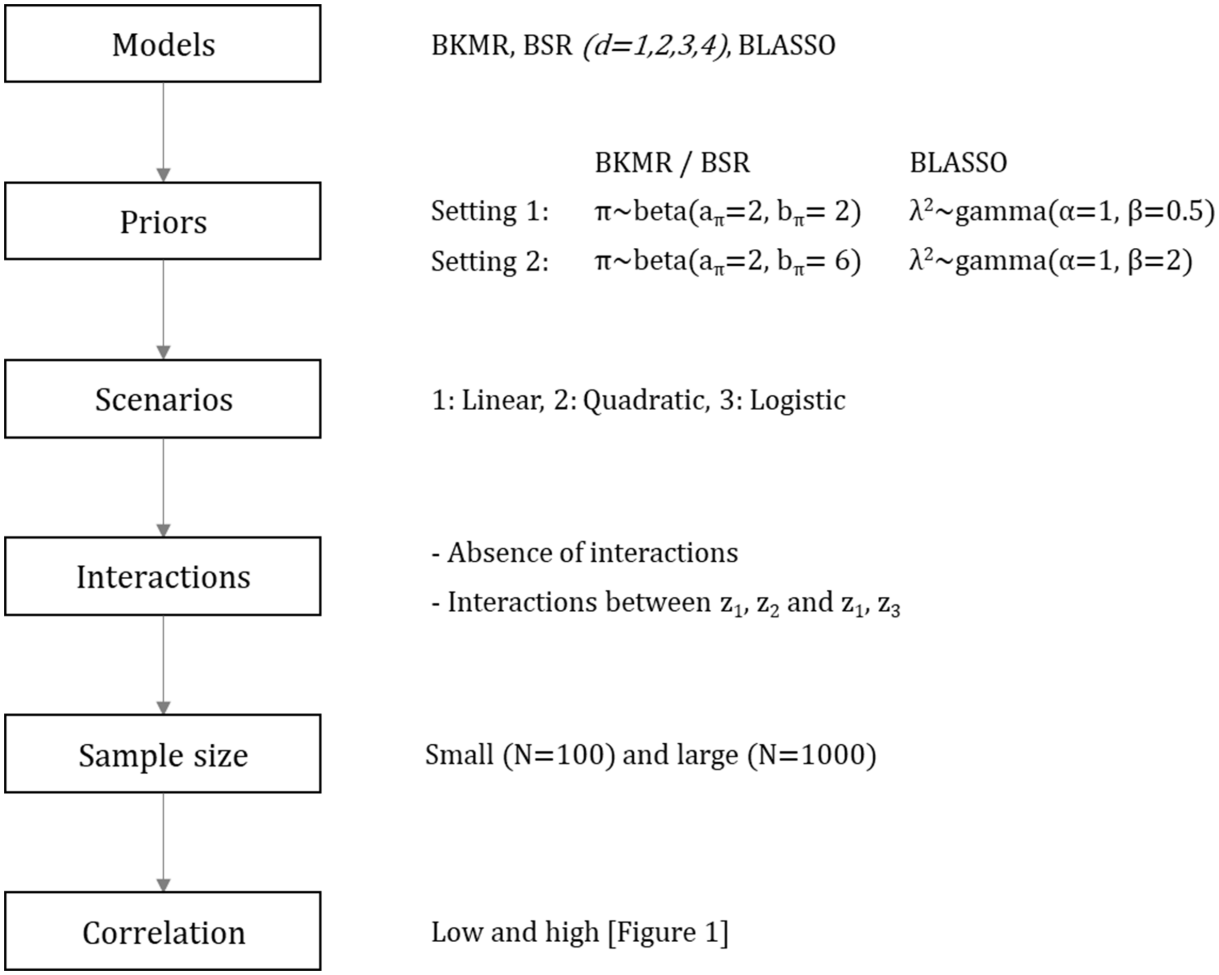
Simulation summary.

For each of these configurations, we generated K = 100 simulated datasets with M = 10 predictors $\left(Z_{1},\ldots ,Z_{10}\right)$ from a multinormal distribution with a mean of zero and a standard deviation of one for each predictor, and with the correlation structure defined above. The predictor-response functions depending on the scenario and the presence/absence of an interaction are shown in Table 1; in Figure 2, a summary of the simulation structure and parameters is presented.

**Table 1:** Predictor-response relationships for the three scenarios.

Scenario 1: linear predictor-response associations	
a)No interactions	$Y_{i}=0.2z_{i1}+1z_{i2}+2z_{i3}+\varepsilon_{i}$
b)With interactions	$Y_{i}=0.2z_{i1}+1z_{i2}+2z_{i3}+z_{i1}z_{i2}+z_{i1}z_{i3}+\varepsilon_{i}$
Scenario 2: quadratic predictor-response associations	
a)No interactions	$Y_{i}=0.2z_{i1}^{2}+1z_{i2}^{2}+2z_{i3}^{2}+\varepsilon_{i}$
b)With interactions	$Y_{i}=0.2z_{i1}^{2}+1z_{i2}^{2}+2z_{i3}^{2}+z_{i1}z_{i2}+z_{i1}z_{i3}+\varepsilon_{i}$
Scenario 3: logistic predictor-response associations	
a)No interactions	$Y_{i}=\frac{e^{\left(0.2z_{i1}+1z_{i2}+2z_{i3}\right)}}{1+e^{\left(0.2z_{i1}+1z_{i2}+2z_{i3}\right)}}+\varepsilon_{i}$
b)With interactions	$Y_{i}=\frac{e^{\left(0.2z_{i1}+1z_{i2}+2z_{i3}+z_{i1}z_{i2}+z_{i1}z_{i3}\right)}}{1+e^{\left(0.2z_{i1}+1z_{i2}+2z_{i3}+z_{i1}z_{i2}+z_{i1}z_{i3}\right)}}+\varepsilon_{i}$
$\varepsilon_{i}\sim N\left(0,1\right)$	

Training and validation sets were selected with an 80/20 ratio for each dataset. We computed the average PIP of each predictor from the training datasets and then reported mean PIPs and standard errors (SEs) for all variables of interest. We fit BSR for a few d values, which are considered optimal in Antonelli et al. (14) (i.e., d ∈ {1, 2, 3, 4}).

We assessed the performance prediction on the validation sets using the mean squared error (MSE), reporting the mean and the standard deviation (SD) for each configuration. To select the important predictors in each model, we used the median probability model (MPM) (17), which consisted of having a marginal PIP of at least 50%.

## Results

**Figure 3:**
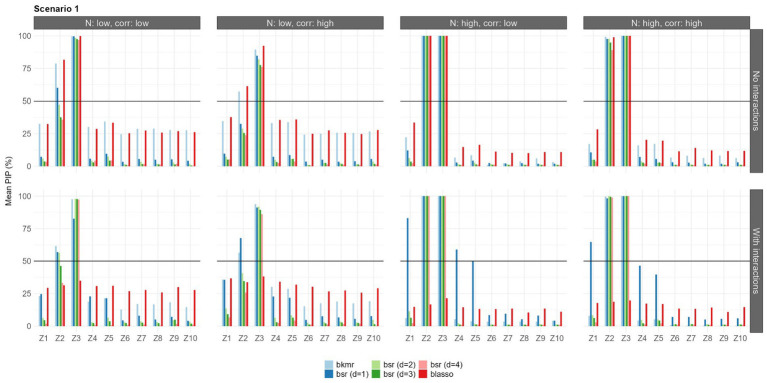
Mean PIPs for each configuration in Scenario 1 (linear predictor-response associations). Results for prior setting 1 shown [π following a beta (a_π_ = 2, b_π_ = 2) for BKMR/BSR, λ^2^ following a gamma (α = 1, β = 0.5) for BLASSO].

**Figure 4:**
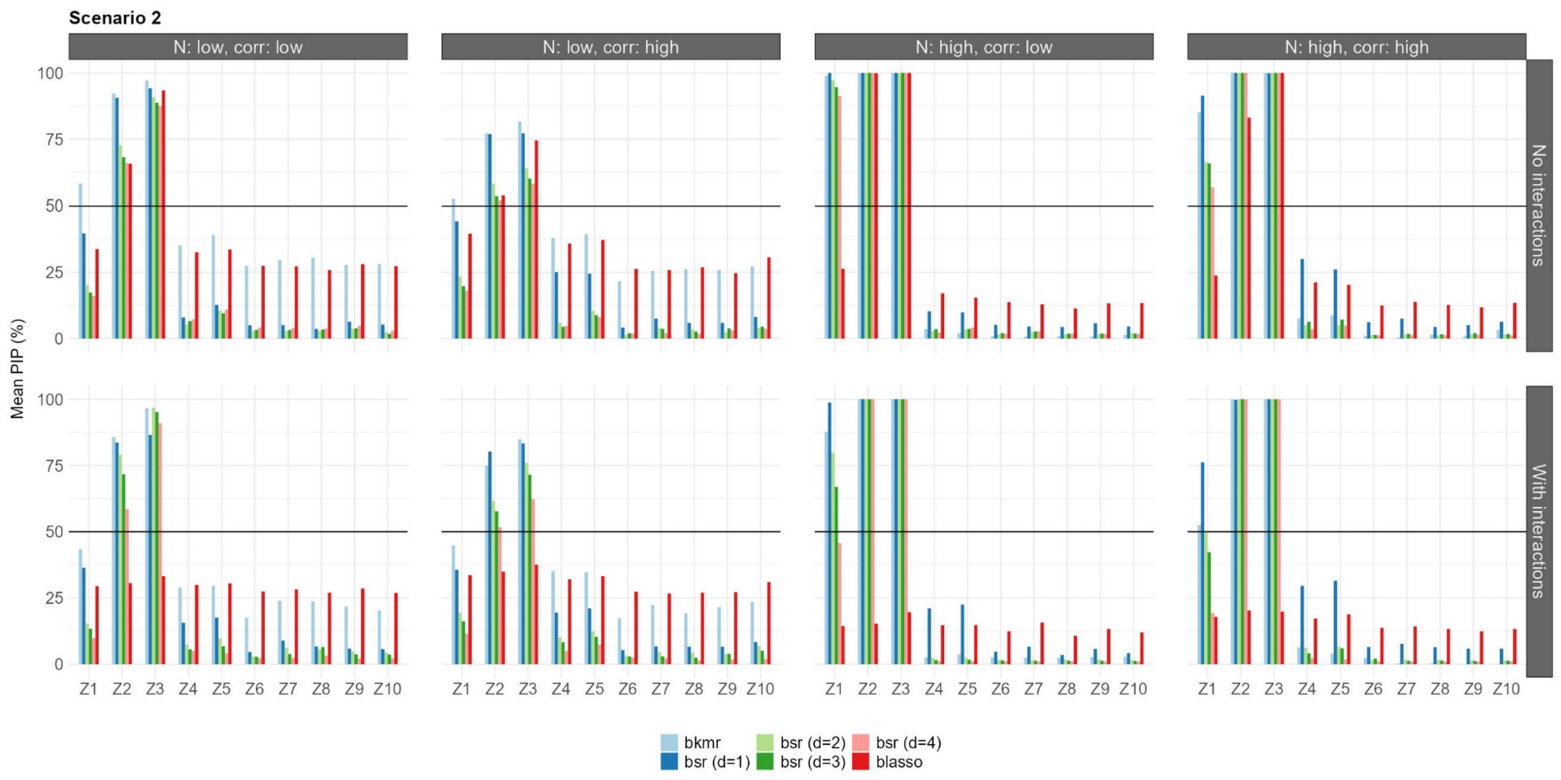
Mean PIPs for each configuration in Scenario 2 (quadratic predictor-response associations). Results for prior setting 1 shown (π following a beta (a_π_ = 2, b_π_ = 2) for BKMR/BSR, λ^2^ following a gamma (α = 1, β = 0.5) for BLASSO].

**Figure 5:**
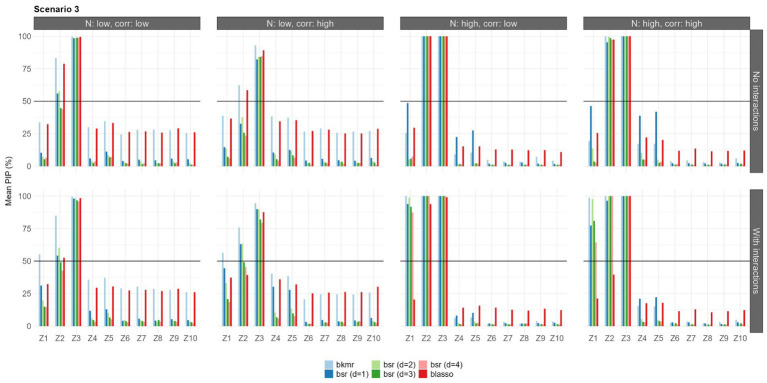
Mean PIPs for each configuration in Scenario 3 (logistic predictor-response associations). Results for prior setting 1 shown (π following a beta (a_π_ = 2, b_π_ = 2) for BKMR/BSR, λ^2^ following a gamma (α = 1, β = 0.5) for BLASSO].

The results in terms of feature selection under each configuration and considering only the first set of priors are shown in Figures 3–5. Due to the robustness of the results, the findings with the second prior setting are shown in Supplementary Figures S1–S3. Each bar represents the mean PIP for each variable and model, and the black horizontal line shows the MPM threshold of 50%.

Under the first scenario, all the models were able to select the predictors $Z_{2}$ and $Z_{3}$ when the sample size was large. With a small sample size (*n* = 100), BLASSO and BKMR showed similar selection abilities, selecting both $Z_{2}$ and $Z_{3}$ . BSR was unable to identify $Z_{2}$ in the situation of high correlation with a sample size of 100. BSR was influenced by the choice of the parameter d; in this scenario, d = 1 led to the best results. $Z_{1}$ was not selected by any model, nor were the highly correlated variables $Z_{4}$ and $Z_{5}$ . Considering the interaction terms, the results were mostly consistent with previous findings, with the exception of the BLASSO, which did not select any predictor. BSR with d = 1 led to the best results when the sample size was large; that is, it showed mean PIP values that were higher than those of the models with different d values. It also identified the weak relationship with $Z_{1}$ , but to the detriment of a greater number of false positives (selecting also the correlated variables $Z_{4}$ and $Z_{5}$ ).

In the second scenario, all the models were able to identify the variables $Z_{2}$ and $Z_{3}\;$ in all configurations. The BKMR and BSR also identified $Z_{1}$ as important in the scenario with a large sample size, while in the low sample size, only the BKMR identified this predictor. When the interactions were included, we detected a decrease in the selection performance of the BLASSO that did not identify any predictors, while the results of the BKMR and BSR were unchanged. BSR showed strong variability in terms of mean PIP as the parameter d varied, especially when the predictor-response relationship was weak.

Finally, in the third scenario, the BKMR was able to select the predictors $Z_{2}$ and $Z_{3}$ in all configurations, both with and without interactions, while the BSR was unable to identify $Z_{2}$ in the scenario with a low sample size, high correlation, and without the interactions. $Z_{1}$ was identified by both models only in when the interactions were included and a large sample size was used, while only the BKMR was able to identify this relationship when the sample size was small. The BLASSO performed well, selecting $Z_{2}$ and $Z_{3}$ in all situations without interactions. When interactions were present, it was able to identify $Z_{3}\;$ and, only with a large sample size and low correlation, also $Z_{2}$ . Changes in prior settings led to consistent results for all models.

To test the predictive ability of the models on the validation set, we used MSE. The results in terms of the mean MSE for each configuration and the first prior setting are shown in Table 2. The results for prior setting 2 are presented in Supplementary Table S1 in our Supplementary material. In all scenarios, the BKMR and BSR showed similar predictive abilities, with comparable mean MSE values. The BLASSO mean MSE values were comparable with the MSE values of the other models only in the situation with no interactions. When the interactions were included, the BLASSO was characterised by higher average MSE values and, therefore, by a lower predictive ability.

**Table 2:** Mean MSE (and SD) for each model for the different simulations’ configurations.

Scenario	Interaction	Sample size	Correlation	BKMR	BSR (d = 1)	BSR (d = 2)	BSR (d = 3)	BSR (d = 4)	BLASSO
1	No	100	Low	7.6 (2.2)	7.5 (2.2)	7.8 (2.1)	8.2 (2.3)	8.4 (2.3)	7.5 (2.1)			High	10.0 (3.3)	9.7 (3.1)	9.9 (3.3)	10.2 (3.4)	10.2 (3.4)	9.7 (3.0)		1,000	Low	7.8 (0.6)	7.6 (1.1)	7.6 (1.1)	7.6 (1.1)	7.7 (1.1)	7.7 (0.8)			High	9.5 (0.8)	9.4 (1.2)	9.4 (1.2)	9.4 (1.2)	9.5 (1.2)	9.4 (1.2)	Yes	100	Low	16.4 (7.1)	19.0 (7.9)	15.7 (6.7)	16.2 (6.5)	16.7 (6.8)	24.8 (11.4)			High	20.5 (9.9)	20.6 (9.1)	19.9 (9.2)	20.3 (9.5)	20.8 (9.9)	30.1 (17.8)		1,000	Low	12.0 (4.4)	16.9 (2.1)	13.2 (1.8)	13.3 (1.8)	13.3 (1.8)	25.3 (3.7)			High	17.4 (1.1)	17.7 (2.3)	17.0 (2.3)	17.1 (2.3)	17.1 (2.3)	33.8 (5.8)
2	No	100	Low	12.8 (4.8)	12.6 (4.2)	13.2 (4.3)	14.5 (5.1)	14.3 (5.2)	14.9 (5.9)			High	17.4 (7.2)	17.3 (7.3)	17.7 (7.4)	18.2 (7.5)	18.5 (7.6)	22.0 (9.6)		1,000	Low	11.2 (1.0)	11.5 (1.6)	11.7 (1.6)	11.9 (1.7)	12.0 (1.7)	15.6 (2.2)			High	15.6 (1.0)	15.5 (2.1)	15.7 (2.1)	15.8 (2.1)	15.9 (1.8)	21.4 (3.0)	Yes	100	Low	36.7 (23.5)	38.9 (23.0)	37.6 (35.0)	37.9 (28.2)	39.1 (24.9)	54.9 (35.5)			High	53.9 (22.1)	52.3 (21.2)	54.7 (24.3)	54.9 (23.8)	57.5 (26.0)	84.7 (44.3)		1,000	Low	28.1 (6.6)	31.6 (3.9)	28.3 (3.6)	28.7 (3.7)	29.3 (3.8)	54.5 (8.6)			High	44.1 (4.2)	43.8 (5.8)	43.2 (5.8)	43.5 (5.8)	43.6 (5.9)	82.9 (13.3)
3	No	100	Low	0.133 (0.044)	0.142 (0.047)	0.143 (0.047)	0.136 (0.046)	0.138 (0.046)	0.141 (0.046)			High	0.147 (0.051)	0.149 (0.052)	0.148 (0.049)	0.145 (0.053)	0.144 (0.051)	0.149 (0.050)		1,000	Low	0.133 (0.013)	0.138 (0.018)	0.134 (0.018)	0.132 (0.017)	0.132 (0.017)	0.143 (0.018)			High	0.143 (0.012)	0.146 (0.019)	0.143 (0.019)	0.141 (0.019)	0.141 (0.019)	0.156 (0.020)	Yes	100	Low	0.133 (0.050)	0.141 (0.054)	0.140 (0.052)	0.137 (0.050)	0.139 (0.048)	0.148 (0.055)			High	0.117 (0.040)	0.120 (0.044)	0.123 (0.048)	0.118 (0.042)	0.121 (0.045)	0.135 (0.044)		1,000	Low	0.118 (0.011)	0.127 (0.016)	0.122 (0.017)	0.120 (0.016)	0.120 (0.017)	0.154 (0.019)			High	0.106 (0.014)	0.118 (0.019)	0.111 (0.020)	0.109 (0.015)	0.108 (0.015)	0.150 (0.020)

Results for prior setting 1 shown [π following a beta (a_π_ = 2, b_π_ = 2) for BKMR/nBSR, λ^2^ following a gamma (α = 1, β = 0.5) for BLASSO].

### Real case study on morbidly obese women

Here we present a case study to compare the models using data from the Istituto Auxologico Italiano. The FUOBAUXO study, approved by the Ethical Committee of the Istituto Auxologico Italiano (protocol research project code: 18A301) (16), included 1,129 women hospitalised for clinical complication care and to access the metabolic rehabilitation program at San Giuseppe Hospital in Piancavallo (VB), Italy. The eligibility criteria for enrolment in this cohort included being over 18 years of age and having a Body Mass Index (BMI) ≥ 30 kg/m^2^. All women were recruited between May 2015 and July 2019. The protocol was explained to the patients, who gave their written informed consent. The overall objective of this study was to determine the association between the rehabilitation program and each patient’s weight loss percentage during the hospitalisation (median length of stay: 35 days, Inter-Quartile Range [IQR]: 33–36 days) and in the 24 months after discharge. The rehabilitation program consisted of a restricted diet (an approximately 15% kcal/day reduction from the resting energy expenditure measure), moderate aerobic physical activity (5 days per week of outdoor walking, 20–30 min cycloergometer, and 1 h aerobic standing exercise), nutritional education, and psychological support (three times per week).

In this study, we focused on the association between several predictors—anthropometric, clinical, and biochemical variables—and weight loss percentage at discharge on obesity-affected women. The predictors included in the models were grouped according to a medical experts’ suggestion in: anthropometric, biochemical, cardiovascular, metabolic, and inflammatory (Supplementary Table S2). Furthermore, predictors were characterised by a complex correlation structure (Supplementary Figure S4). Women with missing information on the predictors were not included in the analyses, thus leaving a total of 755 participants. We divided the initial dataset into training (80%, n_1_ = 604) and validation (20%, n_2_ = 151) sets. Women in the training and validation sets had similar demographic characteristics (Table 3). We fitted the three models on the training dataset and considered the 50% PIP threshold for predictor selection (Table 4). The BSR WAICs when d = 3 and d = 4 were equivalent (2793.0 and 2792.4, respectively). Therefore, we chose the model with d = 3 because the model increases its complexity with an increase of the d value.

**Table 3:** Demographic characteristics of the population: overall population, training set, and validation set.

	Overall (*n* = 755)	Training (*n* = 604)	Validation (*n* = 151)	*p*-value
Age; mean (SD)	58.9 (12.8)	58.9 (13.0)	59.1 (12.4)	0.806^a^
Height; mean (SD)	156.7 (6.8)	156.7 (6.7)	156.6 (7.0)	0.799^a^
BMI; mean (SD)	43.8 (6.7)	43.9 (6.9)	43.2 (6.1)	0.233^a^
Type I (30 ≤ bmi < 35); *n* (%)	40 (5.3)	32 (5.3)	8 (5.3)	0.987^b^
Type II (35 ≤ bmi < 40); *n* (%)	202 (26.8)	161 (26.7)	41 (27.2)	
Type III (bmi ≥ 40); *n* (%)	513 (67.9)	411 (68.0)	102 (67.5)	
Weight at admission; mean (SD)	107.7 (18.9)	108.1 (19.3)	106.0 (16.7)	0.182^a^
Weight at discharge; mean (SD)	102.9 (17.7)	103.2 (18.1)	101.4 (15.8)	0.217^a^
Weight loss; mean (SD)	−4.8 (3.1)	−4.8 (3.2)	−4.6 (2.3)	0.246^a^
Weight loss (%); mean (SD)	−4.4 (2.4)	−4.4 (2.5)	−4.3 (2.0)	0.573^a^

The *p*-value refers to comparisons between training and validation. a = tested by T-test, b = tested by χ^2^ test. The p-value refers to comparisons between training and validation. a = tested by T-test, b = tested by *χ*^2^ test.

**Table 4:** Variables selected, with posterior inclusion probabilities (PIP) ≥ 50%, by the Bayesian kernel machine regression (BKMR), Bayesian semiparametric regression (BSR), and Bayesian Least Absolute Shrinkage and Selection Operator regression (BLASSO) models in explaining weight loss percentage during hospitalization in the FUOBAUXO cohort.

Type	Variable	BKMR	BSR (d = 3)	BLASSO
A	Lean body mass (LBM) (kg)	99.9	81.7	82.6
A	Waist circumference (cm)			59.8
A	Hip circumference (cm)			85.4
A	Body mass index (BMI) (kg/cm^2^)			98.1
A	Heart rate (bpm)			
C	Systolic blood pressure (mmHg)	67.4		
M	Thyroid-stimulating hormone (TSH) (IU/L)	99.3	100.0	
M	Glycated hemoglobin (mmol/mol)			
M	Creatinine (mg/dl)	55.8		
M	Total cholesterol (mg/dl)	54.6		
M	Calcifediol (IU/L)	66.8		
M	Fasting plasma glucose (FPG) (mg/dl)		71.1	
M	Uric acid (mg/dl)			77.2
I	Erythrocyte sedimentation rate (ESR) (mm)	65.9	100.0	
I	C-reactive protein (CRP) (mg/dl)	78.3		

Type: Anthropometric (A), Biochemical (B), Cardiovascular (C), Metabolic (M), Inflammatory (I).

**Figure 6:**
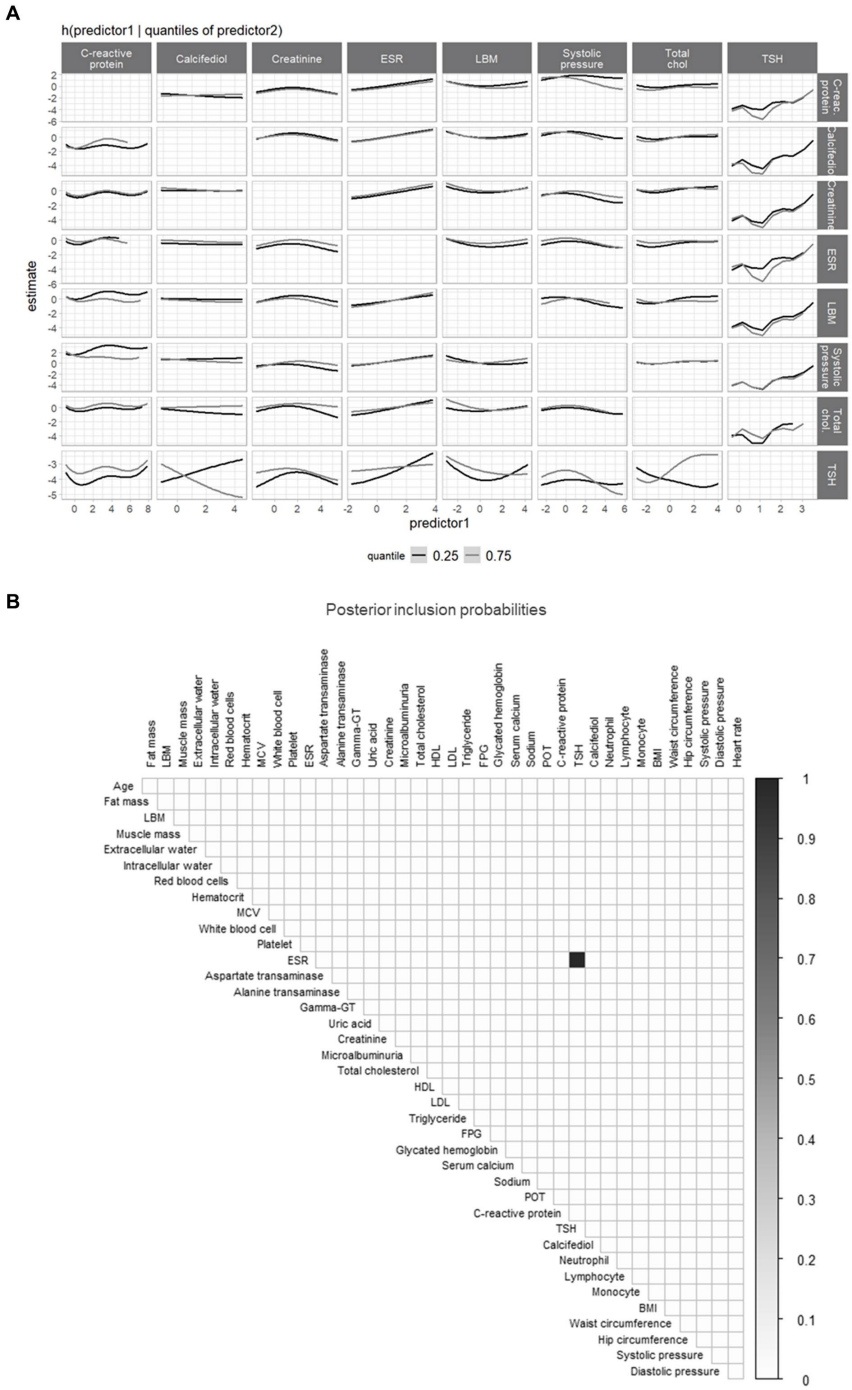
**(A)** Bayesian kernel machine regression (BKMR) interaction plot for the selected predictors, showing the predictor-response function of a single predictor against the second predictor fixed at 0.25 and 0.75 quantiles (remaining predictors are fixed to the median value). **(B)** Heatmap of the posterior inclusion probabilities (PIPs), showing the interactions between the 39 predictors (listed in Supplementary Table S2 of the Supplementary material) identified by Bayesian semiparametric regression (BSR; PIP = 1 between TSH and ESR). LBM, Lean Body Mass; MCV, Mean Corpuscular Volume; ESR, Erythrocyte Sedimentation Rate; Gamma-GT, Gamma-glutamyltransferase; HDL, High Density Lipoprotein; LDL, Low Density Lipoprotein.

In the training data among 39 predictors, 13 were selected by the models. All three models agreed in selecting the lean body mass (mean PIP: BKMR = 99.9%, BSR = 81.7%, BLASSO = 82.7%). However, we identified discrepancies among the variable selections performed by the BKMR, BSR, and BLASSO models. The BLASSO selected all anthropometric predictors except for uric acid. Both the BKMR and BSR selected thyroid-stimulating hormone (TSH) and erythrocyte sedimentation rate, showing a nonadditive effect between the two (BKMR and BSR results are shown in Figure 6). Among the differences between the BKMR and BSR results, we identified fasting plasma glucose, which was a relevant predictor for the BSR but not for the BKMR. The BKMR selected a greater number of variables, suggesting a wider interaction pattern (Figure 6) and more complex mechanisms of action (e.g., between total cholesterol and TSH). We measured and compared the prediction ability of each model by leveraging the validation set and using the MSE. The MSE obtained were 3.6, 7.6, and 3.6. for the BKMR, BSR, and BLASSO, respectively.

## Discussion

In this study, we compared three supervised Bayesian methods (BKMR, BSR, and BLASSO) that were designed to perform variable selection and evaluate the association of multiple predictors on an outcome. We highlighted advantages and limitations of each approach under different data configurations. Both the BKMR and BSR are novel semiparametric approaches that are able to handle nonlinear and nonadditive relationships, while the BLASSO regression accommodates only additive and linear predictor-outcome associations. In the simulations, we observed that the performance of both the BKMR and BSR strongly depends on the sample size and correlation structure.

When the data sample size was large, both the BKMR and BSR correctly selected the predictors associated with the outcome, except when the true relationship was extremely weak. With a small sample size, the predictors more strongly associated with the outcome were always selected by the BKMR. With a small sample size and when the predictor-outcome relationship was weak, only the BKMR correctly selected the predictors, thus making the BKMR the most robust variable selection approach across scenarios. With a small sample size dataset and independently of the strength of predictor-outcome associations, BSR showed high heterogeneity in the variable selection abilities because of strong influence the tuning d parameter. The BLASSO captured well strong linear and additive relationships between predictors and outcome. BLASSO variable selection ability was hampered by strong nonlinear associations or interactions. When there were high correlations between predictors, PIP values were more unstable for all models. In terms of prediction ability, no differences were observed between the BKMR and BSR (for each value of d considered), while the BLASSO seemed to be characterised by higher average MSE values in most configurations. Despite the presence of highly correlated variables that are not associated with the outcome of interest, the models considered do not detect spurious relationships. This confirms their ability to handle situations of high correlation between predictors.

Our case study had a sample size comparable to the large-sample-size synthetic scenarios. When we applied all three models (BKMR, BSR, and BLASSO) to the case study, we observed that, out of all variables, the lean body mass was selected by all models. The selection of at least one anthropometric parameter, such as lean body mass, emphasised the importance of body composition in the process of weight loss. Several weight loss programs have resulted in short-term success, but many patients fail in long-term weight loss maintenance because of the loss of lean body mass that frequently and unintentionally occurs (18). There is a wide literature on the variation of body compartments during weight loss phases, which demonstrates that body composition improvement is mainly associated with the maintenance or increase of lean muscle mass (besides fat mass decrease) when dieting (19). The BLASSO selected other anthropometric measures; however, those variables were supposed to be strongly correlated with lean body mass, thus suggesting that they were false positives.

Both the BKMR and BSR identified the outcome “weight loss” as linked to TSH and the erythrocyte sedimentation rate (ESR), suggesting the existence of a nonlinear relationship between those variables and the outcome. Thyroid hormones have an essential and well-known role in body weight regulation, mainly through energy expenditure modulation. Indeed, hyperthyroidism or hypothyroidism frequently lead to significant changes in body weight (20). Thyroid hormones are tightly linked to cholesterol levels, which may lead to an increase of androgen levels and facilitate weight loss. The ESR is an inflammation marker routinely used in clinical practice to estimate overall inflammation. Common metabolic abnormalities, such as obesity and metabolic syndrome, are pro-inflammatory states that can be associated with increased ESR levels (21). Moreover, in morbidly obese patients, surgically induced weight loss is associated with a marked decrease in ESR and white blood cell count, which may indicate general inflammatory status improvements (22).

The BKMR identified five other variables associated with the outcome, but the BSR did not select them. Based on what we have learnt with the simulated scenarios, those variables may be weakly associated with weight loss, and further studies should investigate the effective role of those variables on weight loss.

In the case study, the BSR identified fasting plasma glucose as associated with weight loss. This variable was correlated with other metabolic variables selected by the BKMR, thus suggesting a connection between metabolic parameters and weight loss. This result was in part expected, given that the beneficial effects of decreased energy intake and weight loss in blood glucose control begin to occur rapidly during rehabilitation programs. The use in the case study of BKMR and BSR, models capable of handling situations with high correlation and complex relationships with the outcome, enabled the identification of important predictors belonging to different clinical groups by selecting only one or a few variables from each group. In contrast, BLASSO selected highly correlated variables largely belonging to a single clinical group, the anthropometric group, with the greatest possibility of identifying spurious relationships between predictors and outcomes.

A few limitations of our study should be noted. Neither the synthetic nor real case scenarios considered the high-dimensional framework, which can include more than 40 predictors and more than 1,000 participants. Moreover, in the simulations, we did not consider departures from the normality assumption in the linear models. Although these approaches adapt very well to high-dimensional biological or clinical contexts with different underlining distribution functions, further studies should assess the performance of those approaches under the more extended framework. Furthermore, considering the role played by the prior choice in the Bayesian variable selection, we selected two prior settings, which guaranteed a low and medium inclusion probability, *a priori*, of each component. However, further studies may need to assess the robustness of the results. Lastly, the real case scenario focused only on Caucasian women undergoing a weight loss program; for this reason, these results cannot be generalised to the entire population, to men, or to minorities who are more prone to develop metabolic conditions (i.e., Hispanics). Larger and more ethnically diverse studies should confirm our findings.

## Conclusion

This is a first attempt to compare the performance of these innovative methods beyond typically environmental contexts and to provide practical usage information. In summary, the performance of all these methods depended on the sample size, the correlation structure, and the relationship between the predictors and the outcome.

The BKMR showed excellent performance in all scenarios, both with high and low correlation and sample size, identifying predictors strongly associated with the outcome and without false positives. It was also able to identify true weak relationships.

The performance of the BSR was also excellent in all different scenarios, but showed a very strong dependence on the choice of its own parameter, especially with a small sample size or high correlations between predictors, showing a higher variability in selection abilities.

The BLASSO performed poorly under nonlinear and synergistic scenarios but had good performance under the linear and additive scenarios. Our results suggest that both the BKMR and BSR may be employed in all scenarios, with attention in selecting an appropriate tuning parameter for BSR when sample size is small. The BLASSO should be preferred when it is possible to hypothesise the absence of interactions between predictors and in the presence of monotonous relationships and low correlations between predictors.

## Data availability statement

The simulation data supporting the conclusions of this article will be made available by the authors, without undue reservation. FUOBAUXO data are available upon request from the Auxologico team. Please contact n.pesenti@campus.unimib.it.

## Ethics statement

The studies involving human participants were reviewed and approved by Ethical Committee of the Istituto Auxologico Italiano (protocol research project code: 18A301). The patients/participants provided their written informed consent to participate in this study.

## Author contributions

NP participated in the conceptualization and design of the work, managed the acquisition of data, gave substantial contributions to the analysis or interpretation of data, wrote the first draft of the paper, gave final approval of the version published, and ensured that questions related to the accuracy or integrity of any part of the work are appropriately investigated and resolved. PQ and EC participated in the conceptualization and design of the work, gave substantial contribution to the interpretation of data, critically revised the work and gave final approval of the version published. RC and MS gave substantial contribution to the interpretation of data, revised the work and gave final approval of the version published. AZ participated in the conceptualization and design of the work, gave essential contribution to the interpretation of data, revised the draft giving important intellectual contribution and gave final approval of the version published and ensured that questions related to the accuracy or integrity of any part of the work are appropriately investigated and resolved. All authors contributed to the article and approved the submitted version.

## Funding

During the preparation of this manuscript, EC was supported by the National Institute of Environmental Health Science (NIEHS): R01ES032242 and P30ES023515.

## Conflict of interest

The authors declare that the research was conducted in the absence of any commercial or financial relationships that could be construed as a potential conflict of interest.

## Publisher’s note

All claims expressed in this article are solely those of the authors and do not necessarily represent those of their affiliated organizations, or those of the publisher, the editors and the reviewers. Any product that may be evaluated in this article, or claim that may be made by its manufacturer, is not guaranteed or endorsed by the publisher.
