# Supplementary material for: Comparative efficacy of three Bayesian variable selection methods in the context of weight loss in obese women
Source: Front Nutr. 2023 Jul 18;10:1203925. doi: 10.3389/fnut.2023.1203925 (PMC10390836; doi:10.3389/fnut.2023.1203925)
Supplement: Supplementary file 1 [file Data_Sheet_1.docx]

**Comparative efficacy of three Bayesian variable selection methods in the context of weight loss in obese women
(Supplemental material)**

Nicola Pesenti^1^, Piero Quatto^2^, Elena Colicino^3^, Raffaella Cancello^4^, Massimo Scacchi^5,6^, and

Antonella Zambon^1,7^

^1^ Department of Statistics and Quantitative Methods, Division of Biostatistics, Epidemiology and Public Health, University of Milano-Bicocca, Milan, Italy.

^2^ Department of Economics, Management and Statistics, University of Milano-Bicocca, Milan, Italy.

^3^ Department of Environmental Medicine and Public Health, Icahn School of Medicine at Mount Sinai, New York, NY 10029, USA.

^4^ Obesity Unit and Laboratory of Nutrition and Obesity Research, Department of Endocrine and Metabolic Diseases, IRCCS Istituto Auxologico Italiano, Milan, Italy.

^5^ Division of Endocrinology and Metabolic Diseases, IRCCS Istituto Auxologico Italiano, 28824 Piancavallo (VB), Italy.

^6^ Department of Clinical Science and Community Health, University of Milan, 20100 Milan, Italy.

^7^ Istituto Auxologico Italiano, IRCCS, Biostatistic Unit, Milan, Italy.

**Corresponding author:**

Nicola Pesenti, Department of Statistics and Quantitative Methods, Division of Biostatistics, Epidemiology and Public Health, University of Milano-Bicocca, 20126, Milan, Italy.

Email: [n.pesenti@campus.unimib.it](mailto:n.pesenti@campus.unimib.it)

Figure S1. Mean PIPs for each configuration in Scenario 1 (linear predictor-response associations). Results for prior setting 2 shown (π following a beta(a_π_=2, b_π_=6) for BKMR/BSR, λ^2^ following a gamma(α=1, β=2) for BLASSO).


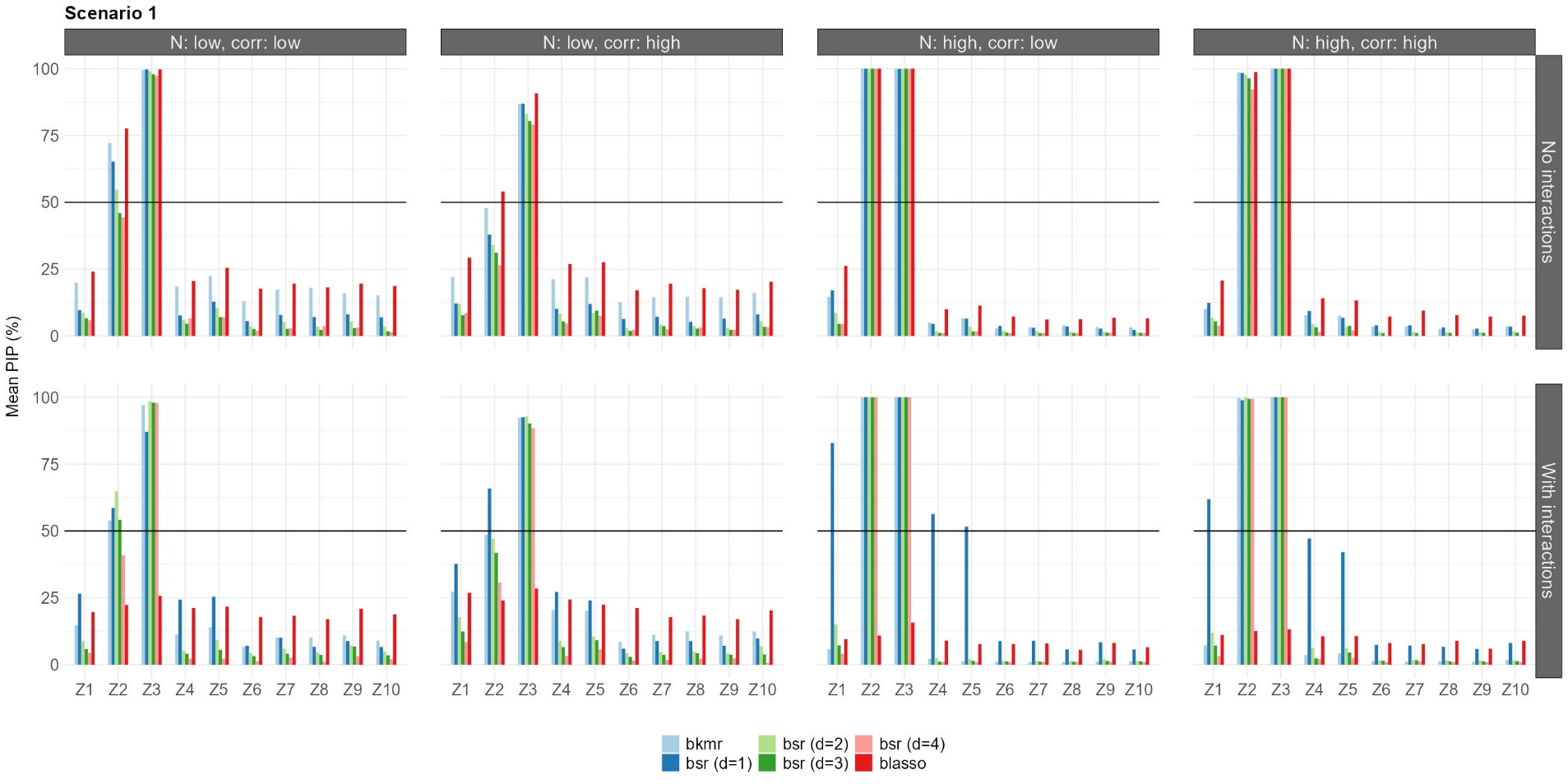


Figure S2. Mean PIPs for each configuration in Scenario 2 (quadratic predictor-response associations). Results for prior setting 2 shown (π following a beta(a_π_=2, b_π_=6) for BKMR/BSR, λ^2^ following a gamma(α=1, β=2) for BLASSO).


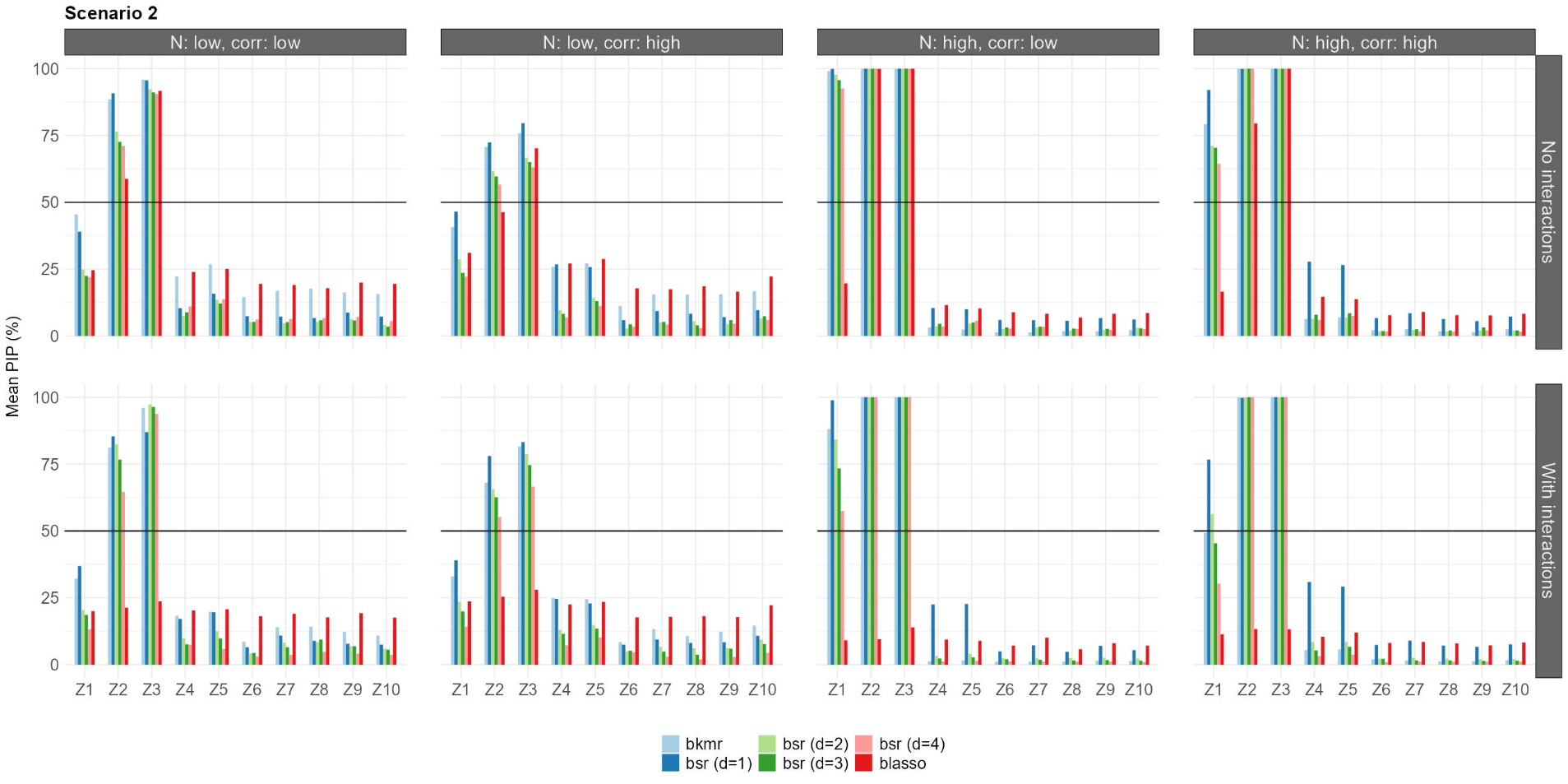


Figure S3. Mean PIPs for each configuration in Scenario 3 (Logistic predictor-response associations). Results for prior setting 2 shown (π following a beta (a_π_=2, b_π_=6) for BKMR/BSR, λ^2^ following a gamma(α=1, β=2) for BLASSO).


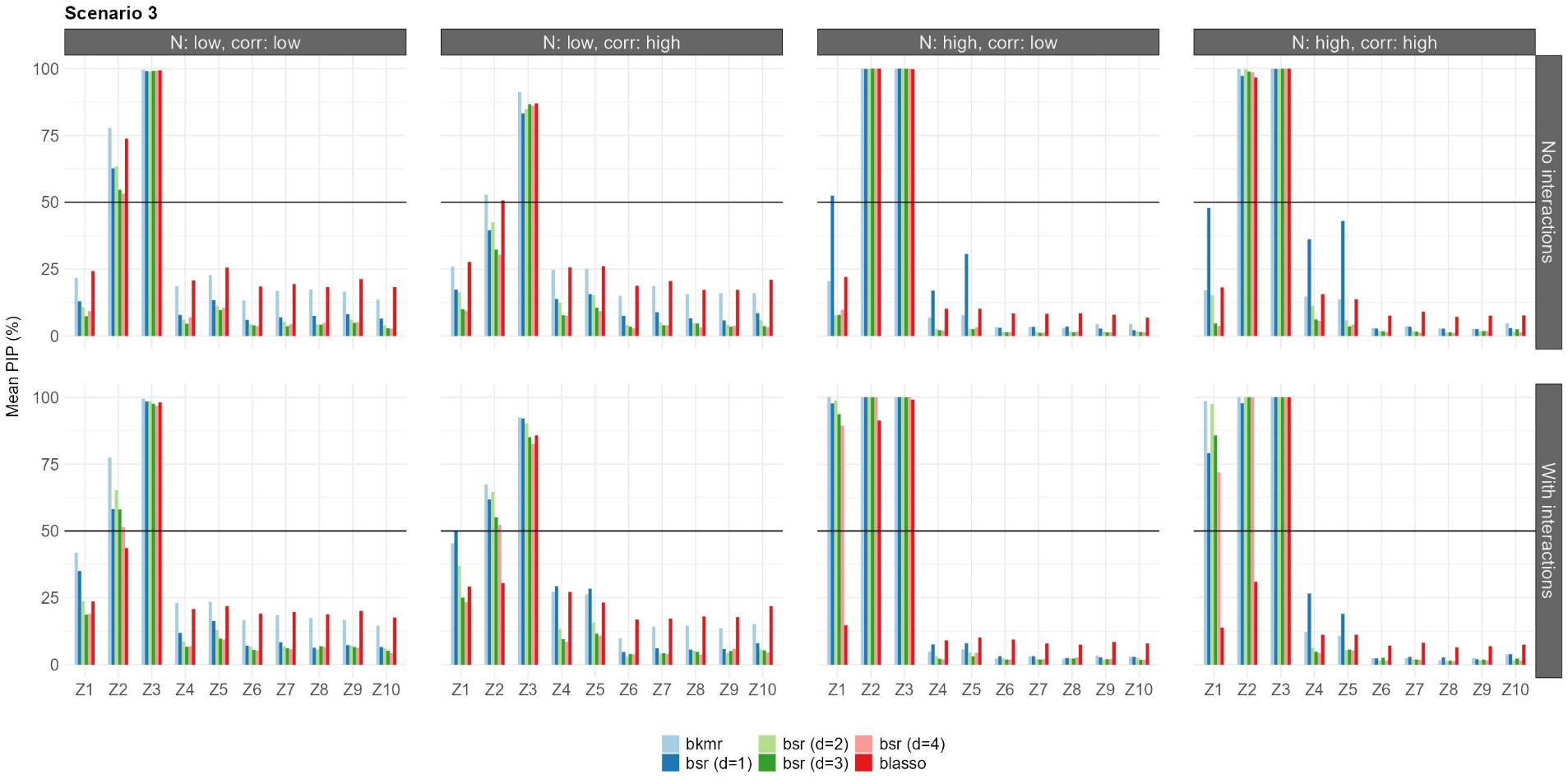


*Table S1. Mean MSE (and SD) for each model for the different simulations’ configurations. Results for prior setting 2 shown (π following a beta (a_π_=2, b_π_=6) for BKMR/BSR, λ^2^ following a gamma(α=1, β=2) for BLASSO).*

| **Scenario** | **Interaction** | **Sample size** | **Correlation** | **BKMR** | **BSR (d=1)** | **BSR (d=2)** | **BSR (d=3)** | **BSR (d=4)** | **BLASSO** |
| --- | --- | --- | --- | --- | --- | --- | --- | --- | --- |
| 1 | No | 100 | Low | 7.5 (2.2) | 7.5 (2.2) | 7.7 (2.2) | 8.1 (2.3) | 8.4 (2.4) | 7.5 (2.1) |
|  |  |  | High | 9.8 (3.2) | 9.7 (3.1) | 9.9 (3.4) | 10.2 (3.4) | 10.3 (3.4) | 9.7 (3.0) |
|  |  | 1000 | Low | 7.6 (1.1) | 7.6 (1.1) | 7.6 (1.1) | 7.6 (1.1) | 7.7 (1.1) | 7.7 (0.8) |
|  |  |  | High | 9.4 (1.2) | 9.4 (1.2) | 9.4 (1.2) | 9.4 (1.2) | 9.5 (0.8) | 9.4 (1.2) |
|  | Yes | 100 | Low | 16.2 (7.0) | 19.1 (7.8) | 15.6 (6.7) | 15.9 (6.4) | 16.6 (6.8) | 24.7 (11.4) |
|  |  |  | High | 20.4 (9.6) | 20.7 (9.0) | 19.9 (9.2) | 20.3 (9.5) | 20.7 (9.8) | 30.0 (17.6) |
|  |  | 1000 | Low | 13.3 (1.8) | 16.9 (2.1) | 13.2 (1.8) | 13.2 (1.8) | 13.2 (1.8) | 25.3 (3.7) |
|  |  |  | High | 17.1 (2.3) | 17.7 (2.3) | 17.0 (2.3) | 17.1 (2.3) | 17.1 (2.3) | 33.7 (5.8) |
| 2 | No | 100 | Low | 12.6 (4.6) | 12.6 (4.3) | 13.1 (4.2) | 14.3 (4.7) | 14.2 (5.0) | 14.9 (5.9) |
|  |  |  | High | 17.6 (7.4) | 17.3 (7.1) | 17.7 (7.4) | 18.1 (7.5) | 18.4 (7.5) | 22.0 (9.6) |
|  |  | 1000 | Low | 11.6 (1.6) | 11.5 (1.6) | 11.7 (1.6) | 11.9 (1.7) | 12.0 (1.7) | 15.6 (2.2) |
|  |  |  | High | 15.6 (2.2) | 15.5 (2.1) | 15.7 (2.1) | 15.8 (2.2) | 15.9 (2.2) | 21.4 (3.0) |
|  | Yes | 100 | Low | 35.7 (21.3) | 38.6 (22.3) | 37.8 (34.2) | 38.0 (29.2) | 38.9 (24.0) | 54.7 (35.5) |
|  |  |  | High | 54.5 (23.8) | 52.3 (21.0) | 54.8 (23.3) | 54.4 (22.7) | 56.8 (24.8) | 84.3 (44.3) |
|  |  | 1000 | Low | 28.2 (3.6) | 31.6 (3.9) | 28.3 (3.6) | 28.7 (3.7) | 29.3 (4.0) | 54.5 (8.6) |
|  |  |  | High | 43.5 (5.9) | 43.8 (5.8) | 43.2 (5.8) | 43.6 (5.8) | 43.7 (5.9) | 82.9 (13.3) |
| 3 | No | 100 | Low | 0.133 (0.044) | 0.141 (0.046) | 0.143 (0.047) | 0.135 (0.046) | 0.137 (0.045) | 0.141 (0.046) |
|  |  |  | High | 0.144 (0.050) | 0.148 (0.053) | 0.148 (0.050) | 0.144 (0.053) | 0.144 (0.050) | 0.149 (0.050) |
|  |  | 1000 | Low | 0.131 (0.017) | 0.138 (0.018) | 0.134 (0.018) | 0.132 (0.017) | 0.133 (0.017) | 0.143 (0.018) |
|  |  |  | High | 0.140 (0.019) | 0.145 (0.019) | 0.143 (0.019) | 0.141 (0.019) | 0.141 (0.019) | 0.156 (0.020) |
|  | Yes | 100 | Low | 0.132 (0.051) | 0.140 (0.054) | 0.141 (0.052) | 0.136 (0.050) | 0.139 (0.049) | 0.148 (0.055) |
|  |  |  | High | 0.114 (0.037) | 0.121 (0.046) | 0.123 (0.045) | 0.116 (0.042) | 0.121 (0.045) | 0.135 (0.043) |
|  |  | 1000 | Low | 0.118 (0.016) | 0.127 (0.016) | 0.122 (0.017) | 0.120 (0.017) | 0.120 (0.017) | 0.154 (0.019) |
|  |  |  | High | 0.106 (0.014) | 0.118 (0.019) | 0.111 (0.020) | 0.109 (0.015) | 0.108 (0.015) | 0.150 (0.020) |

Table S2. List of anthropometric, biochemical, cardiovascular, metabolic, and inflammatory variables considered.

| **Type** | **Variable** | **Type** | **Variable** |
| --- | --- | --- | --- |
| A | Age (years) | M | Low Density Lipoprotein (LDL) (mg/dL) |
| A | Fat mass (FM) (kg) | M | Triglyceride (mg/dL) |
| A | Lean body mass (LBM) (kg) | M | Fasting plasma glucose (FPG) (mg/dL) |
| A | Muscle mass (kg) | M | Glycated hemoglobin (mmol/mol) |
| A | Extracellular water (%) | M | Serum calcium (mg/dL) |
| A | Intracellular water (%) | M | Sodium (mmol/L) |
| A | Waist circumference (cm) | M | Potassium (POT) (mmol/L) |
| A | Hip circumference (cm) | M | Uric acid (mg/dL) |
| A | Body mass index (BMI) (kg/cm^2^) | M | Thyroid-stimulating hormone (TSH) (IU/L) |
| B | Red blood cells (IU/L) | M | Calcifediol (IU/L) |
| B | Hematocrit (%) | I | White blood cell (K/uL) |
| B | Mean corpuscular volume (MCV) (μm^3^) | I | Erythrocyte sedimentation rate (ESR) (mm) |
| C | Platelet (IU/L) | I | Aspartate transaminase (AST) (IU/L) |
| C | Heart rate (bpm) | I | Alanine transaminase (ALT) (IU/L) |
| C | Systolic blood pressure (mmHg) | I | Gamma-glutamyltransferase (GGT) (IU/L) |
| C | Diastolic pressure (mmHg) | I | C-reactive protein (CRP) (mg/dL) |
| M | Creatinine (mg/dL) | I | Neutrophil (AGC) (IU/L) |
| M | Microalbuminuria (mg) | I | Lymphocyte (IU/L) |
| M | Total cholesterol (mg/dL) | I | Monocyte (IU/L) |
| M | High Density Lipoprotein (HDL) |  |  |

*Note: Type: Anthropometric (A), Biochemical (B), Cardiovascular (C), Metabolic (M), Inflammatory (I), Other (O).*


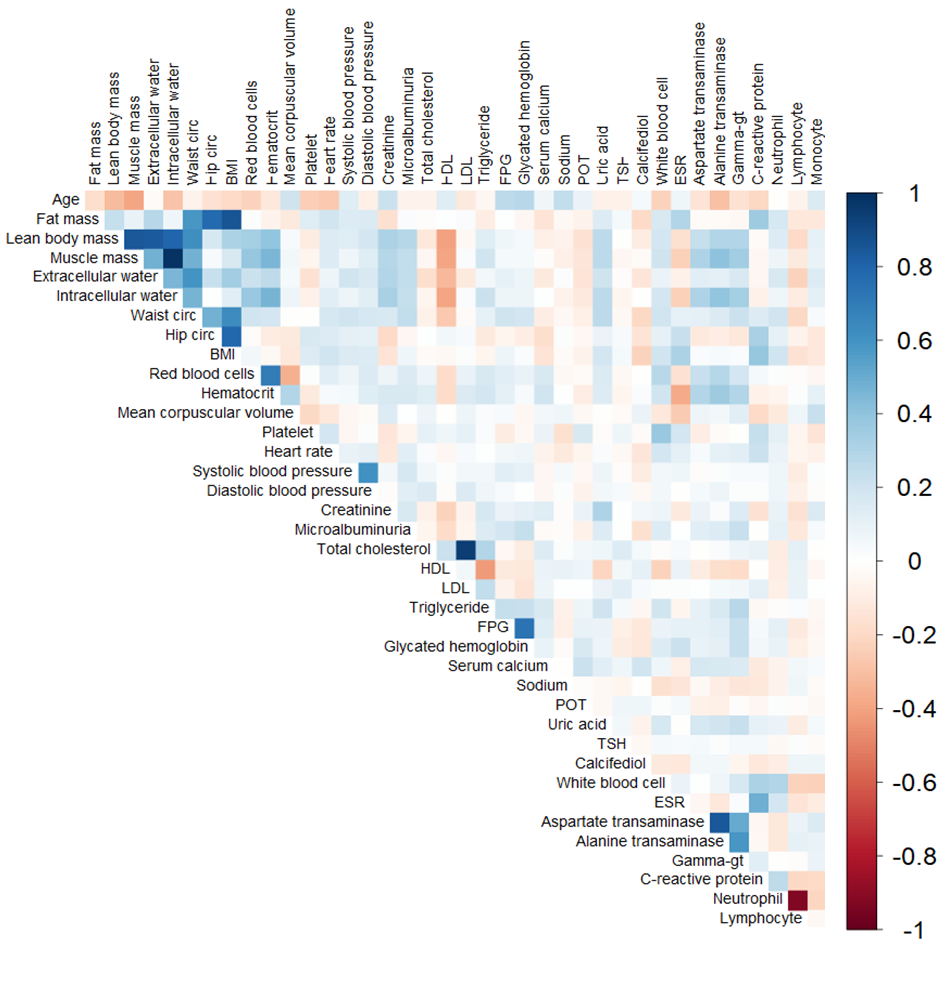
Figure S4. Spearman’s correlation matrix between all the variables considered.
